# Evaluation of Browning Agents on the White Adipogenesis of Bone Marrow Mesenchymal Stromal Cells: A Contribution to Fighting Obesity

**DOI:** 10.3390/cells10020403

**Published:** 2021-02-16

**Authors:** Girolamo Di Maio, Nicola Alessio, Ibrahim Halil Demirsoy, Gianfranco Peluso, Silverio Perrotta, Marcellino Monda, Giovanni Di Bernardo

**Affiliations:** 1 Human Physiology and Unit of Dietetic and Sports Medicine Section, Department of Experimental Medicine, School of Medicine, University of Campania Luigi Vanvitelli, 80138 Naples, Italy; girolamo.dimaio@yahoo.it (G.D.M.); marcellino.monda@unicampania.it (M.M.); 2 Department of Experimental Medicine, Biotechnology and Molecular Biology Section, School of Medicine, University of Campania Luigi Vanvitelli, 80138 Naples, Italy; nicola.alessio@unicampania.it (N.A.); ibrahimdemirsoy@gmail.com (I.H.D.); 3 Institute Bioscience and BioResources, CNR, 80131 Naples, Italy; gianfranco.peluso@cnr.it; 4 Dipartimento della Donna, del Bambino e di Chirurgia Generale e Specialistica, School of Medicine, Università degli Studi della Campania Luigi Vanvitelli, 80131 Naples, Italy; silverio.perrotta@unicampania.it

**Keywords:** mesenchymal stromal cells, adipogenesis, BAT, WAT, browning

## Abstract

Brown-like adipocytes can be induced in white fat depots by a different environmental or drug stimuli, known as “browning” or “beiging”. These brite adipocytes express thermogenin UCP1 protein and show different metabolic advantages, such as the ability to acquire a thermogenic phenotype corresponding to standard brown adipocytes that counteracts obesity. In this research, we evaluated the effects of several browning agents during white adipocyte differentiation of bone marrow-derived mesenchymal stromal cells (MSCs). Our in vitro findings identified two compounds that may warrant further in vivo investigation as possible anti-obesity drugs. We found that rosiglitazone and sildenafil are the most promising drug candidates for a browning treatment of obesity. These drugs are already available on the market for treating diabetes and erectile dysfunction, respectively. Thus, their off-label use may be contemplated, but it must be emphasized that some severe side effects are associated with use of these drugs.

## 1. Introduction

Overweight and obesity are described by the World Health Organization (WHO) as aberrant or superfluous fat accumulation that is dangerous for health due to their association with chronic multifactorial conditions (e.g., type 2 diabetes, some types of cancers, and cardiovascular diseases); they cause a great burden in terms of healthcare costs [1,2,3,4].

For a long time, adipose tissue was considered as a passive energy storage area; only at the end of the previous century was it identified as the body’s principal “factory”, able to manufacture various hormones and adipokines (signaling molecules) which play a fundamental action in food intake, energy homoeostasis, insulin sensitivity, angiogenesis, and blood pressure [5]. Consequently, it is considered an endocrine organ that plays a crucial role in metabolism [6]. Most of these properties can be ascribed to white adipose tissue (WAT), which represents a deposit of energy overbalance as triglycerides in a single lipid droplet that invade the majority of the adipocytes’ volume and a few mitochondria scattered along the nucleus border [7]. In contrast, brown adipocytes present in brown adipose tissue (BAT) are involved in glucose and lipid homeostasis as well as in modulating body temperature, spreading energy by producing heat via UCP1 protein (uncoupling protein 1), which uncouples substrate oxidation from ATP synthesis. Brown adipocytes contain a high number of mitochondria and multiple lipid droplets [8,9].

**Figure 1:**
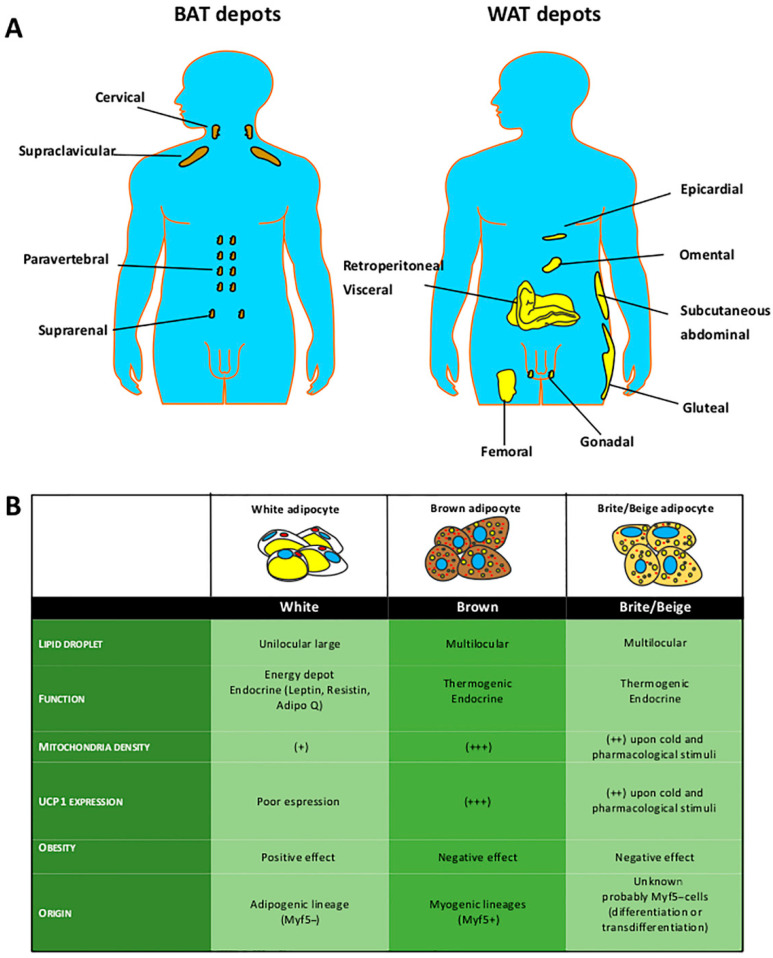
White, brown, and brite/beige adipose depots. (**A**) Body distribution of subcutaneous (gluteal, abdominal, and femoral), visceral (gonadal, mesenteric, epicardial, omental, and retroperitoneal), and brown (paravertebral, supraclavicular, and suprarenal) adipose tissue depots in a human model. BAT: brown adipose tissue; WAT: white adipose tissue. (**B**) Comparison among white, brown, and brite/beige adipose tissue.

WAT is present in every part of the body (Figure 1) and is often categorized into visceral (vWAT) and subcutaneous (sWAT) storage, depending on the location. In humans, BAT depots are far less abundant than WAT, and are concentrated in well-defined anatomical positions (the supraclavicular, cervical, paravertebral, perirenal, and mediastinal regions). BAT depots decrease with age [10,11].

Multidisciplinary strategies are fundamental to counteracting obesity. Weight-loss approaches range from changing eating habits (e.g., diets, weight-loss programs) to more drastic strategies, such as endoscopic or bariatric surgery. The latter represents an alternative choice, especially for when healthy diet and physical activity changes are inadequate or fail. While weight-loss surgery offers the best chance of losing weight, it can pose serious risks. With some approaches, in addition to caloric restrictions and standard gym regimens, a prescription for specific weight loss medications can be useful [12,13]. Nevertheless, in some cases, most of these strategies fail to induce weight loss in overweight and obese patients.

Alternative approaches have been proposed to counteract obesity. Many studies have shown that activation of brown adipogenesis can contribute to energy expenditure, suppressing obesity [14,15,16]. Several strategies have been implemented to stimulate brite/beige adipogenesis in WAT, including cold exposure and pharmacological and plant agent treatments. The induction of brown-like adipocytes in WAT is called “browning” or “beiging” [17], and is closely associated with the expression of the UCP1 protein that governs thermogenesis [18,19].

In this research, we evaluated the effects of several browning agents during white adipocyte differentiation of mesenchymal stromal cells (MSCs), which are able to differentiate both in brown and white adipocytes [20,21]. MSCs are present within WAT depots. These cells contain several sub-populations, which comprise multipotent stem cells (able to produce adipocytes osteocytes and chondrocytes), fibroblasts, stromal cells, and pro-genitor cells [22,23]. Our in vitro findings identified two compounds that may warrant further in vivo investigation as possible anti-obesity drugs.

## 2. Materials and Methods

### 2.1. MSC Cultures

The experimental procedures were performed according to the rules approved by the Ethics Committee of the Luigi Vanvitelli Campania University (887/2008). Patients were informed about the current research and gave permission for the use of MSCs.

Cells were separated on a Ficoll density gradient (GE Healthcare, Milan, Italy), and the mononuclear cell fraction was collected and washed in PBS 1X (EuroClone, Milan, Italy). Approximately 1–2.5 × 10^5^ cells/cm^2^ were seeded in an alpha-MEM (EuroClone, Italy) containing 10% FBS (EuroClone, Italy) and 3 ng/mL of β-FGF (Prepotech, London, UK). After 72 h, non-adherent cells were discarded, and adherent cells were further cultivated to confluency. MSC cultures were controlled in order to satisfy the three proposed criteria for defining MSCs [24].

At P3, MSCs were used for treatment with different drugs—1μM FGF-21 (Prepotech, UK) [25], 20nM Glucagon-Like Peptide 1 (GLP-1) (Sigma-Aldrich, Saint Louis, MO, USA) [26], 1nM GW501516 (Sigma-Aldrich, USA), 1nM sildenafil (SID) (Sigma-Aldrich, Saint Louis, MO, USA) [27], or 43nM Rosiglitazone (ROSI) (Sigma-Aldrich, Saint Louis, MO, USA)—and incubated for 48 h or 21 days, as indicated in the results.

### 2.2. Cell Cycle Analysis

For every assay, cells were fixed and collected in 70% ethanol for O.N. at −20 °C, then washed with PBS 1X, and then dissolved in a hypotonic buffer containing propidium iodide. The assays were performed on a Guava EasyCyte flow cytometer (Merck Millipore, Burlington, MA, USA) and analyzed with a standard procedure using EasyCyte software.

### 2.3. In Situ Senescence-Associated ß-Galactosidase Assay

A solution of 2% formaldehyde and 0.2% glutaraldehyde was used to fix the cells. After that, cells were washed with PBS and then incubated at 37 °C for at least 18 h with a staining solution [citric acid/phosphate buffer (pH 6), K4Fe(CN)6, K3Fe(CN)6, NaCl, MgCl2, X-Gal. Blue cells represented the number of b-galactosidase-positive cells out of at least 500 cells in a different microscope field; this number was used to calculate the percentage of senescent cells, as already reported [28]. All reagents were obtained from (Sigma-Aldrich, Saint Louis, MO, USA).

### 2.4. Annexin-V Assay

A Guava EasyCyte (Merck Millipore, Burlington, MA, USA) flow cytometer was used to evaluate apoptotic cells using a kit of fluorescein conjugated with Annexin V, following the manufacturer’s instructions. Apoptotic and non-apoptotic cells were analyzed and identified by two separate dyes (Annexin V and 7AAD) to cover a broad spectrum of cells: phosphatidylserine was bound by Annexin V (green) on the external membrane of apoptotic cells, while 7AAD (red) permeated and stains DNA in late-stage apoptotic and dead cells. Coloration enabled the identification of 3 cell populations: non-apoptotic cells (Annexin V- and 7AAD+); early apoptotic cells (annexin V+ and 7AAD-); and late apoptotic or dead cells (Annexin V+ and 7AAD+). In our experimental conditions, both the early apoptotic and late apoptotic cells were grouped.

### 2.5. White and Brown Differentiation

White adipogenic differentiation was conducted as such: MSCs at 70–80% confluence had the medium replaced with an adipogenic induction medium composed of high-glucose DMEM (EuroClone, Milan, Italy) and supplemented with 10% FBS (EuroClone, Napoli, Italy), 1% penicillin/streptomycin (EuroClone, Italy), 1 mM dexamethasone (Sigma-Aldrich, USA), 10 µg/mL insulin (Sigma-Aldrich, Saint Louis, MO, USA), 0.5mM 3-isobutyl-1-methylxanthine (Sigma-Aldrich, USA), and 200 µM indomethacin (Sigma-Aldrich, USA). Cells were cultured in this medium for 21 days.

Brown adipogenic differentiation was conducted as such: MSCs were cultured in the same condition as white adipogenic differentiation for 14 days. After that, the medium was changed to DMEM/F12 (EuroClone, Milan, Italy) and supplemented with 10% FBS, 200 uM of ascorbic acid (Sigma-Aldrich, Saint Louis, MO, USA), 20 nM of insulin, 0.2 nM of T3 (Sigma-Aldrich, Saint Louis, MO, USA) and 1 uM od b-adrenoreceptor agonist CL316243 (Sigma-Aldrich, Saint Louis, MO, USA). The cells were cultivated for another 7 days.

The differentiation took place in the presence or absence of drugs, as indicated in the results.

### 2.6. Oil Red O Staining

Oil Red O staining was used to confirm the adipogenic differentiation, which is an indicator of intracellular lipid accumulation. The differentiated cultures were washed with PBS and then fixed with 4% formaldehyde for ten minutes at room temperature (RT). Cells were subsequently washed with isopropanol (3%) and treated with the Oil Red O staining solution. Stained cultures were analyzed under a light microscope [24]. All reagents were obtained from (Sigma-Aldrich, Saint Louis, MO, USA).

### 2.7. RNA Extraction and RT-qPCR

TRIREAGENT (Molecular Research Center Inc., USA) was used to extract RNA from cell cultures. To quantify the mRNA levels by real-time PCR amplification, a process already reported [29], mRNA sequences were retrieved from a nucleotide data bank (National Center for Biotechnology Information) in order to design primer pairs for RT-PCR reactions (Primer Express, Applied Biosystems, Foster City, CA, USA). As controls, appropriate regions of GAPDH mRNA were used. Real-time PCR assays were carried out with an Opticon 4 machine (MJ Research, St. Bruno, QC, Canada); reactions were performed according to the manufacturer’s instructions. An SYBR green PCR master mix was used, and the 2-ΔΔCT method was employed as a relative quantification method.

### 2.8. Mitochondrial ATP Production and Proton Leak 

The measurement of mitochondrial ATP and proton leak was performed with MitoXpress® Xtra assay (Luxcel Biosciences Cork, Ireland). This assay uses an oxygen-sensing fluorophore that is quenched by O_2_ through molecular collision; as a result, the amount of fluorescence signal is inversely proportional to the amount of extracellular O_2_ in the sample. First, we measured the basal respiration; subsequently, oligomycin and FCC were added sequentially, which targeted the components of the electron transport chain (ETC) in the mitochondria, which allowed us to determine the mitochondrial ATP production. Then, we added antimycin A, which blocks Q coenzyme, and established the proton leak [30].

### 2.9. UCP1 Immunocytochemistry

We identified lipid droplets with BODIPY staining. Live cells were incubated with 10 µM green, fluorescent fatty acid, BODIPY 505/515 (Invitrogen—Thermofisher Scientific, MS, USA) for 30 min in DMEM-free serum at 37 °C in the culture incubator. Then, cells were washed with PBS, fixed with 2% formaldehyde for 15 min at RT, and washed with PBS. The UCP1 (sc518024, Santa Cruz, USA) was detected in the cell cultures according to the manufacturer’s instructions. Cell nuclei were stained with 4′,6-diamidino-2-phenylindole (DAPI) and then observed with a fluorescence microscope (Leica Wetzlar, Germany). We calculated the percentage of UCP1-positive cells by counting a minimum of 500 cells in different microscope fields.

### 2.10. Statistical Analysis

Statistical significance was evaluated with ANOVA, followed by the Student’s *t*-test or Bonferroni’s test. A mixed-model variance analysis was used for data with continuous outcomes. Data were evaluated with a GraphPad Prism version 5.01 statistical software package (GraphPad, La Jolla, CA, USA).

## 3. Results

### 3.1. Browning Drugs Did Not Affect MSC Biological Parameters

**Figure 2:**
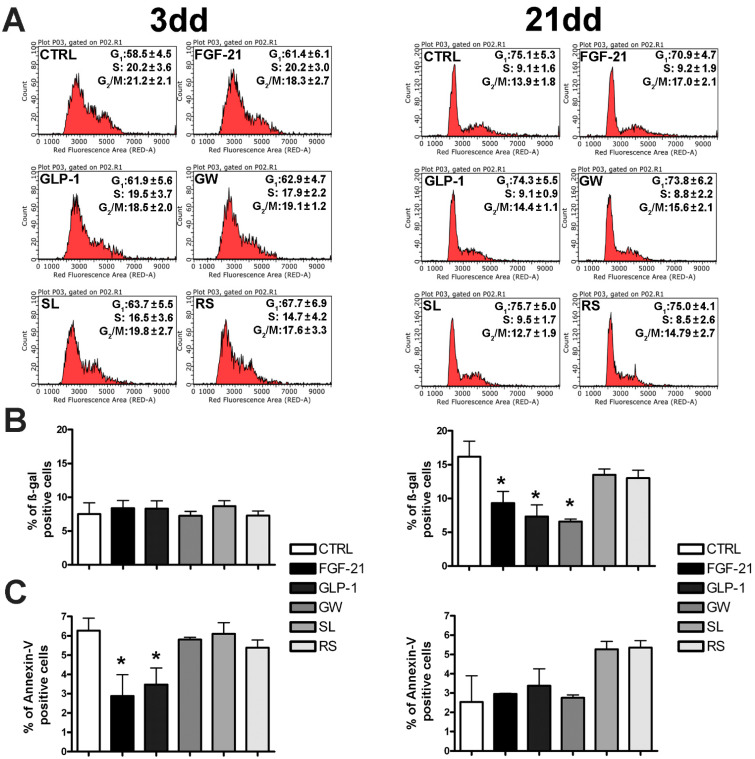
Cell growth, senescence, and apoptosis evaluation. (**A**) The picture shows the cell cycle profiles of mesenchymal stromal cells (MSCs) incubated with drugs and compared with controls (CTRL) following three and 21 days of treatment (± SD, *n* = 5 biological replicates). (**B**) Senescence analysis (following three and 21 days of treatment) by 4-methylumbelliferyl-β-d-galactopyranoside (MUG) quantitative fluorescent assay. A β-galactosidase substrate, 4-MUG only emits fluorescence when it is cleaved by the enzyme to generate fluorophore 4-methylumbelliferone. The fluorophore production was monitored at an emission/excitation wavelength of 365/460 nm. The data are expressed as arbitrary units (± SD, *n* = 5 biological replicates; * *p* < 0.05). (**C**) Apoptosis detection following three (left) and 21 (right) days of treatment by the fluorescein-conjugated Annexin V assay. Apoptotic cells were identified with a fluorescence microscope. The graph shows mean expression values in MSCs and in CTRLs (± SD, *n* = 5 biological replicates; * *p* < 0.05). FGF-21: fibroblast growth factor-21; GLP-1: glucagon-like peptide-1; GW: GW501516; SL: sildenafil; RS: rosiglitazone. For browning agent details, refer to Table 1.

MSC cell cultures isolated in the bone marrow of healthy donors were used to evaluate whether some in vitro biological properties were differently affected by incubation with browning agents (Table 1). Cell cycle profiles of MSCs incubated with drugs did not differ significantly from those of controls following both three and 21 days of treatment (Figure 2A). In addition, the senescence process was also unaffected by drugs after three days of treatment, while 21 days of FGF-21 or GLP-1 or GW501516 treatment reduced the senescence compared with controls (Figure 2B). In control cultures, apoptosis and necrosis rates were very low and remained modest in the presence of drugs, even if some statistical differences were detected (Figure 2C).

**Figure 3:**
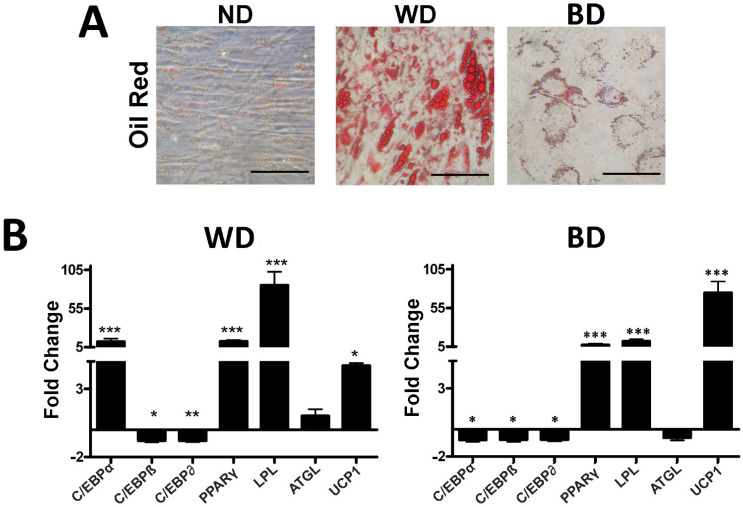
Lipid droplets and gene expression analysis of adipose differentiation markers. Panel **A**: evaluation of lipid droplets’ size by Oil Red O staining in mesenchymal stromal cells (MSCs). Scale bar: 100 µm. Panel **B**: histogram shows RT-qPCR analysis, mRNA expression levels of *C/EBPα*, *C/EBPβ*, *C/EBPδ*, *PPARγ*, *LPL*, *ATGL*, and *UCP1* MSCs in white differentiation and brown differentiation. *GAPDH* mRNA was used as an internal control. Data are expressed as fold change using the delta–delta CT method (± SD, *n* = 5 biological replicates; * *p* < 0.05, ** *p* < 0.01, *** *p* < 0.001). ND: no differentiation cues; WD: white differentiation; BD: brown differentiation.

We induced MSC differentiation in white and brown adipocytes and evaluated the expression levels of early and late differentiation genes involved in adipogenesis. This experiment was performed in order to provide a reference expression profile, allowing us to ascertain whether browning agents can influence white adipogenesis. In the experimental conditions, white adipocytes showed an increase in the genes *C/EBPα* and *PPARγ* and a very significant upregulation (more than 100-fold) of *LPL*. The *ATGL* late gene was also increased (Figure 3A). The brown adipocytes showed an increase in *PPARγ* and *LPL* expression. Importantly, they expressed high levels of UCP1 (Figure 3B). In addition, in brown adipocytes, the *C/EBPα* expression was almost undetectable. This experiment demonstrated that, in our system, *C/EBPα* is almost exclusively expressed in white adipocytes and UCP1 is predominant in brown adipocytes.

### 3.2. Effect of Browning Agents on White Adipogenesis

**Figure 4:**
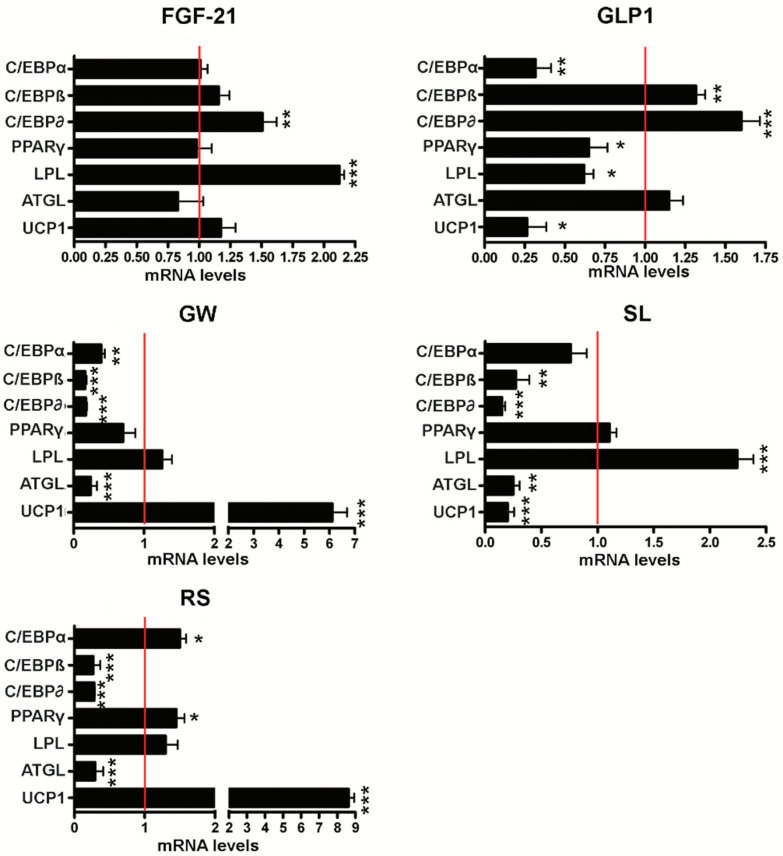
Gene expression analysis of adipose differentiation markers. RT-qPCR analysis of *C/EBPα*, *C/EBPβ*, *C/EBPδ*, *PPARγ*, *LPL*, *ATGL*, and *UCP1* in MSCs treated with browning agents during white adipogenic differentiation. *GAPDH* mRNA was used as an internal control. Data are expressed as fold change using the delta–delta CT method (± SD, *n* = 5 biological replicates; * *p* < 0.05, ** *p* < 0.01, *** *p* < 0.001). FGF-21: fibroblast growth factor-21; GLP-1: glucagon-like peptide-1; GW: GW501516; SL: sildenafil; RS: rosiglitazone. For browning agent details, refer to Table 1.

We then induced white adipogenic differentiation in the presence of drugs to evaluate their browning/beiging effect. Rosiglitazone is a well-known browning agent [31,32]; as expected, it induced a strong upregulation of *UCP1* and a significant decrease in *C/EBPα*, along with increased *PPARγ* levels (Figure 4). The FGF-21 did not modify either *UCP1* or *C/EBPα* mRNA levels compared to the control: it induced an upregulation of *LPL* and C/EBPδ (Figure 4). GW501516 promoted UCP1 upregulation and a decrease in all the C/EBP factors and of ATGL. The other two compounds we tested (GLP-1 and sildenafil) induced a decline in *UCP1* and *C/EBPα*, with some other modifications (mainly in C/EBPβ, δ, and *LPL*) (Figure 4).

These data appeared to indicate that the analyzed drugs were ineffective in browning. Nevertheless, some studies have shown that during browning treatment, the expression levels of UCP1 mRNA and the corresponding protein are not synchronous and may diverge [33]. This consideration—along with the observation that some of the analyzed agents induced a downregulation of *C/EBPα*, as in the rosiglitazone treatment—prompted us to better evaluate the browning effect of the selected compounds by UCP1 protein detection with immunocytochemistry.

**Figure 5:**
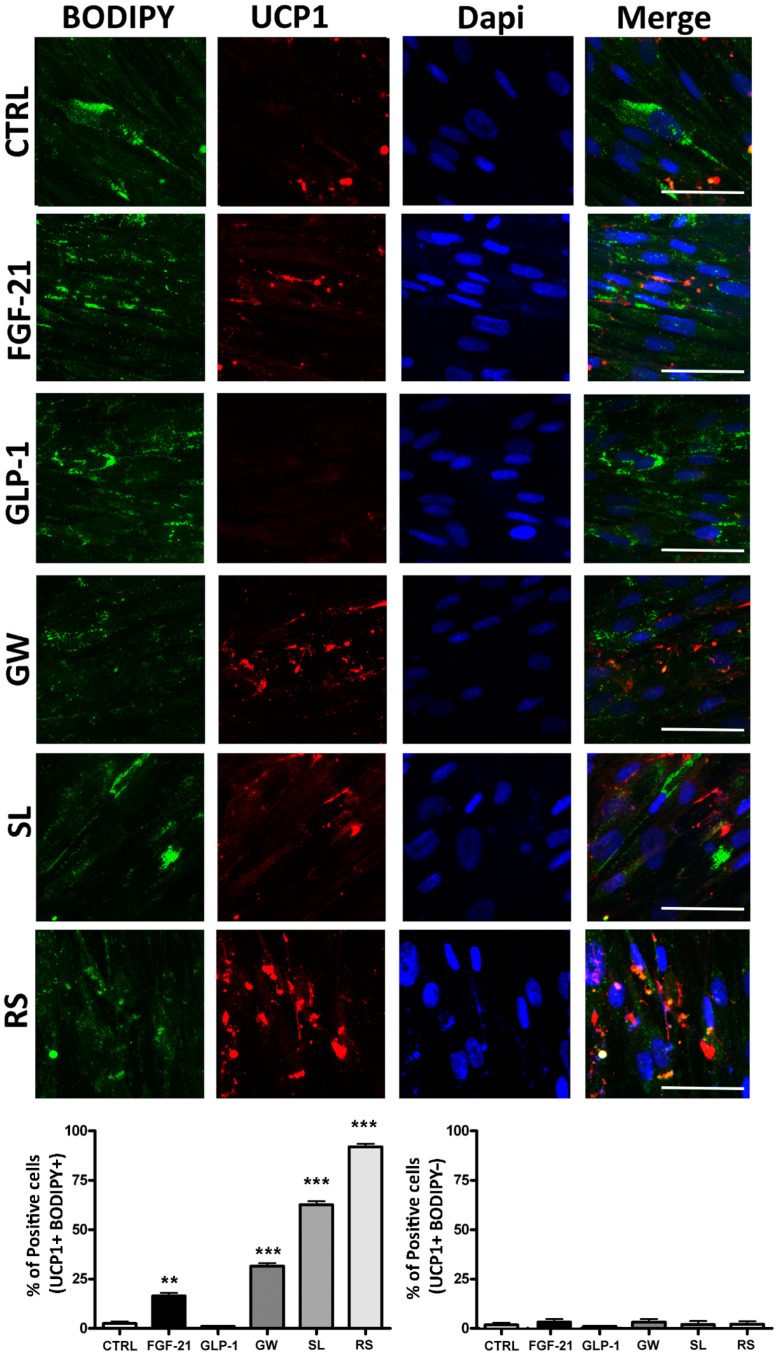
Expression of thermogenin UCP1 protein by immunocytochemistry analysis in MSCs treated with browning agents during white adipogenic differentiation. Cells were counterstained with DAPI and BODIPY 505/515 for the identification of nuclei and lipid depots, respectively. Scale bar: 100 µm. The histograms reveal the percentage of UCP1-positive cells by counting a minimum of 500 cells in different microscope fields (± SD, *n* = 5 biological replicates; ** *p* < 0.01, *** *p* < 0.001). The left graph shows the percentage of UCP1 and BODIPY-positive cells. The right graph indicates the percentage of UCP1-positive and BODIPY-negative cells. CTRL: control; FGF-21: fibroblast growth factor-21; GLP-1: glucagon-like peptide-1; GW: GW501516; SL: sildenafil; RS: rosiglitazone. For browning agent details, refer to Table 1.

We detected a UCP1 protein in 14.2% of BODIPY-positive cells present in cultures treated with FGF-21 compared to the 3.0% observed in control cultures (Figure 5). The GW501516 and sildenafil induced a highly significant increase in the percentage of UCP1-positive cells (Figure 5). These values were detected in cells that accumulated lipid droplets, as demonstrated with BODIPY staining. This result may suggest that increase in UCP1 expression occurred in adipocytes and/or pre-adipocytes.

### 3.3. Functional Evaluation of UCP1 Following Treatments

**Figure 6:**
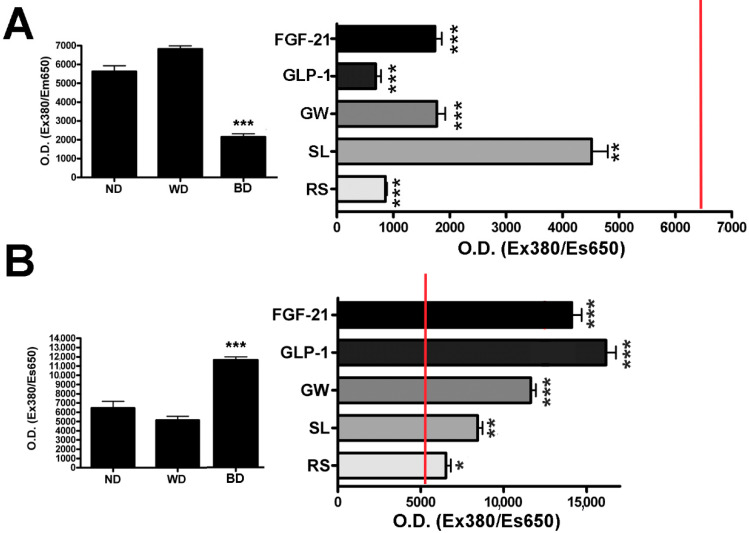
Evaluation of cellular mitochondrial function by oxygen consumption assay. (**A**) Histogram on the left show ATP production in white adipocytes (white differentiation, WD) compared to MSCs grown without differentiation cues (ND). Histogram on right shows mitochondrial ATP production in presence of all tested compounds during white adipogenic differentiation. (**B**) Histogram on the left shows proton leak level in white adipocytes (WD) compared to MSCs grown without differentiation cues (ND). Histogram on right shows the proton leak level in the presence of all tested compounds during white adipogenic differentiation. (± SD, *n* = 5 biological replicates; * *p* < 0.05, ** *p* < 0.01, *** *p* < 0.001). ND: no differentiation cues; WD: white differentiation; BD: brown differentiation; FGF-21: fibroblast growth factor-21; GLP-1: glucagon-like peptide-1; GW: GW501516; SL: sildenafil; RS: rosiglitazone. For browning agent details, refer to Table 1.

The UCP1 protein uncouples oxidative phosphorylation from ATP synthesis, by modifying the internal mitochondrial membrane permeability with a proton leak increment that reduces the ATP yield of cellular respiration in order to dissipate energy as heat [30]. Therefore, we evaluated the cellular mitochondrial function by oxygen consumption assay. We focused our attention on two mitochondrial functional parameters: ATP production and proton leak. Brown adipocytes showed reduced ATP production and increased proton leak compared to white adipocytes and undifferentiated MSCs (Figure 6A,B left graphs). In the presence of all tested compounds, the mitochondrial ATP production was reduced compared to white adipocytes; this phenomenon is associated with an apparent proton leak increment due to increased UCP1 activity (Figure 6A,B, right graphs).

## 4. Discussions

Great advancements have been achieved in detecting new signaling pathways and stimuli that make brown-like adipocytes in white fat depots in order to obtain metabolic advantages, such as combatting obesity and increasing energy expenditure. As a result, an emerging class of pharmacological compounds has been tested and named “browning agents” [34,35,36]. We evaluated different browning agents in vitro to determine the most effective agents that could pave the way for in vivo experimentation.

As a transcription factor, PPARγ represents the cellular target of anti-diabetic thiazolidinedione drugs such as rosiglitazone [37]. Activation of PPARγ and PPAR-response elements (PPREs) can promote expression of the genes involved in browning/beiging [38,39]; rosiglitazone activates thermogenic gene expression and browning phenomena in WAT [37,40]. In our study, the rosiglitazone treatment induced the activation of the transcription factors PPARγ and C/EBPs (β and δ) and increased the gene expression of LPL and UCP1 (Figure 4). The expression of the C/EBPα gene, which is required for white differentiation, was silenced [41]. These data support the role of rosiglitazone as a valid browning agent that can promote the transformation of WAT in brown-like adipose tissue. It cannot be excluded that rosiglitazone or some of the other administered drugs had a significant influence on the differentiation rate of the adipocytes. Otherwise, UCP1 might be induced in preadipocytes in response to certain treatments, as has was been reported [42].

FGF-21, a component of the superfamily of FGF proteins, is involved in glucose and lipid metabolism, and is critical in thermoregulation by promoting energy expenditure. In diabetes and obesity treatment, FGF-21 may play a role in browning induction. We detected a partial browning activity of FGF-21, with 14.2% of white adipocytes appearing to shift toward a brown phenotype, as indicated by UCP1 expression (Figure 5), reduced mitochondrial ATP production, and increased proton leak. In FGF-21-treated cultures, we also observed a strong LPL increment. Notably, in a mouse animal model which has been reported in the literature, the exogenous administration of FGF-21 enhanced LPL activity in peripheral organs such as skeletal muscle and brown fat depots, in which stored fatty acids are transformed into triglyceride-rich lipoproteins [43,44,45]. Thus, the LPL increase is important in metabolizing lipoproteins.

GLP-1, through its receptor GLP-1R, promotes insulin production and glucagon reduction release, and it may stimulate brown fat thermogenesis [46]. Thus, GLP-1 is considered a promising therapeutic approach for the treatment of obese type 2 diabetic patients [16]. In our experimental model, GLP-1 treatment did not produce significant browning effects—we detected a downregulation of UCP1 expression both at the RNA and at the protein levels; however, GLP-1-treated cells showed a reduction in ATP production and a proton leak increment. These physiological data may be explained by the idea that GLP-1 may impair cell metabolic functions without inducing browning—indeed, proton leak has also been associated with pathological conditions [30].

GW501516 is a PPARδ receptor agonist that entered clinical experimentation as a drug for metabolic and cardiovascular diseases [47]. GW501516 seemed to have a higher browning effect than FGF-21 and GLP-1—in white adipocyte differentiation, we detected a C/EBPα mRNA decrement, increased percentage of UCP1-positive cells (32%), reduction in ATP production, and increased proton leak. Although these are promising results, this compound must be carefully utilized, because it may promote cancer development [48].

Sildenafil acts via a nitric oxide (NO)-cyclic guanosine monophosphate (cGMP) pathway to promote penis erectile activity. However, this drug may also have a role in obesity treatment, because activation of the NO/cGMP pathway is important to brown adipocyte development [49]. Sildenafil produced the most promising results besides rosiglitazone: we observed a strong C/EBPα mRNA decrement and a very significant increase in UCP1-positive cells (64%), reduction in ATP production, and increase in proton leak. It should be emphasized that the increase in the percentage of UCP1-positive cells was not associated with upregulation of the corresponding mRNA. There are studies evidencing that UCP1 mRNA and the related protein do not follow the same expression profile [33]. In this context, there are findings showing that only one-third of mRNA levels significantly correlate with proteins [50]. While endogenous or exogenous browning drugs represent an inviting and innovative therapeutic strategy to ameliorate metabolic diseases such as diabetes and obesity, it is important to also weigh the possible side-effects in order for its administration to constitute a health benefit.

## 5. Conclusions

In conclusion, we found that rosiglitazone and sildenafil are the most promising drug candidates for a browning treatment for obesity. These drugs are already available on the market for the treatment of diabetes and erectile dysfunction, respectively. Thus, their off-label use may be contemplated, but it must be emphasized that some severe side effects are associated with the use of these drugs. For example, rosiglitazone and sildenafil may increase the risk of strokes and heart attacks [51,52,53]. Thus, our future investigation will conduct careful in vivo experimentation on the browning effect of these drugs, along with in-depth analysis of the possible side effects.

## Figures and Tables

**Table 1:** Browning agents as anti-obesity treatments.

Drug	Mechanism
GLP-1	Glucose stimulation of insulin secretion by pancreatic β-cells
rosiglitazone	PPAR gamma agonist
GW501516	PPAR delta agonist
FGF-21	Cell-surface FGF receptor activator signaling
Sildenafil	cGMP-dependent protein kinase I and mechanistic/mammalian target of rapamycin (mTOR) signaling pathways

GLP-1: glucagon-like peptide-1; GW501516: 2-[2-methyl-4-[[[4-methyl-2-[4-(trifluoromethyl)phenyl]-5-thiazolyl]methyl]thio]phenoxy]-acetic acid; FGF-21: fibroblast growth factor-21.
